# The Quest for Neurodegenerative Disease Treatment—Focusing on Alzheimer’s Disease Personalised Diets

**DOI:** 10.3390/cimb45020098

**Published:** 2023-02-09

**Authors:** Matei Palimariciuc, Ioana-Miruna Balmus, Bogdan Gireadă, Alin Ciobica, Roxana Chiriță, Alin-Constantin Iordache, Mihai Apostu, Romeo Petru Dobrin

**Affiliations:** 1 Department of Medicine III, Faculty of Medicine, Grigore T. Popa University of Medicine and Pharmacy of Iasi, 16 Universității Street, 700115 Iasi, Romania; 2 Institute of Psychiatry “Socola”, 36 Bucium Street, 700282 Iasi, Romania; 3 Department of Exact Sciences and Natural Sciences, Institute of Interdisciplinary Research, Alexandru Ioan Cuza University of Iasi, Alexandru Lapusneanu Street, No. 26, 700057 Iasi, Romania; 4 Department of Biology, Faculty of Biology, Alexandru Ioan Cuza University of Iasi, B dul Carol I, No. 11, 700506 Iasi, Romania; 5 Academy of Romanian Scientists, Splaiul Independentei nr. 54, Sector 5, 050094 Bucuresti, Romania; 6 Centre of Biomedical Research, Romanian Academy, B dul Carol I, No. 8, 700506 Iasi, Romania; 7 Faculty of Medicine, Grigore T. Popa University of Medicine and Pharmacy of Iasi, 16 Universitatii Strada, 700115 Iasi, Romania; 8 Faculty of Pharmacy, Grigore T. Popa University of Medicine and Pharmacy of Iasi, 16 Universității Street, 700115 Iasi, Romania

**Keywords:** Alzheimer’s disease, dementia, nutritional psychiatry, neuroprotection, oxidative stress, Mediterranean diet, selenium, *Ginkgo biloba*, *Panax ginseng*, *Curcuma longa*

## Abstract

Dementia represents a clinical syndrome characterised by progressive decline in memory, language, visuospatial and executive function, personality, and behaviour, causing loss of abilities to perform instrumental or essential activities of daily living. The most common cause of dementia is Alzheimer’s disease (AD), which accounts for up to 80% of all dementia cases. Despite that extensive studies regarding the etiology and risk factors have been performed in recent decades, and how the current knowledge about AD pathophysiology significantly improved with the recent advances in science and technology, little is still known about its treatment options. In this controverted context, a nutritional approach could be a promising way to formulate improved AD management strategies and to further analyse possible treatment strategy options based on personalised diets, as Nutritional Psychiatry is currently gaining relevance in neuropsychiatric disease treatment. Based on the current knowledge of AD pathophysiology, as well as based on the repeatedly documented anti-inflammatory and antioxidant potential of different functional foods, we aimed to find, describe, and correlate several dietary compounds that could be useful in formulating a nutritional approach in AD management. We performed a screening for relevant studies on the main scientific databases using keywords such as “Alzheimer’s disease”, “dementia”, “treatment”, “medication”, “treatment alternatives”, “vitamin E”, “nutrition”, “selenium”, “*Ginkgo biloba*”, “antioxidants”, “medicinal plants”, and “traditional medicine” in combinations. *Results*: nutrients could be a key component in the physiologic and anatomic development of the brain. Several nutrients have been studied in the pursuit of the mechanism triggered by the pathology of AD: vitamin D, fatty acids, selenium, as well as neuroprotective plant extracts (i.e., *Ginkgo biloba*, *Panax ginseng*, *Curcuma longa*), suggesting that the nutritional patterns could modulate the cognitive status and provide neuroprotection. The multifactorial origin of AD development and progression could suggest that nutrition could greatly contribute to the complex pathological picture. The identification of adequate nutritional interventions and the not yet fully understood nutrient activity in AD could be the next steps in finding several innovative treatment options for neurodegenerative disorders.

## 1. Introduction

Alzheimer’s disease is the most prevalent progressive neurodegenerative disease affecting almost 30% of people 85 years and older [1]. While it is currently estimated that 43–75% of dementia cases are diagnosed as AD [2,3], and there are around 50 million AD patients worldwide, a recent estimation forecasted that the prevalence would double every 5 years and will increase to reach 152 million by 2050 [4]. In this context, the burden of AD patients on their families, medical system, economy, and society could become unbearable.

Despite that extensive and thorough studies regarding the etiology and risk factors were performed in recent decades and the current knowledge about AD pathophysiology greatly improved with the scientific and technological boom, the treatment options are still based on symptomatologic relief and less on disease progression modulation [5,6,7,8]. In this way, the current AD therapies comprise mainly cholinesterase inhibitors and N-methyl-D-aspartate receptor antagonists that could both provide enhanced quality of life by improving AD-nondependent physiological processes rather than disease progression inhibition [9]. However, their mechanisms of action and short- versus long-term effects are extremely controverted [10,11,12]. Only the newest member of the FDA-approved AD therapy squad, aducanumab, seems to provide better results in disease progression modulation by immune-targeting Aβ (beta-amyloid) deposits, but serious issues regarding their effectiveness and safety are currently putting this innovative monoclonal antibody therapy on halt [13,14]. Similarly, numerous clinical trials based on single-agent therapy failed to modulate disease progression, suggesting that the complex and multifactorial pathophysiology of AD could need combined therapy rather than monotherapy.

In this controverted context, a nutritional approach could be a promising way to formulate improved AD management strategies and to further analyse possible treatment options based on personalised diets, as Nutritional Psychiatry is currently gaining relevance in neuropsychiatric disease treatment. For instance, previous reports indicated that higher adherence to Mediterranean diets could be associated with decreased cognitive decline and reduced incidence of AD in the elderly [15,16]. This could be due to both its plant-based plan and due to the increased content of valuable nutrients, such as fibres, good quality fats and lipid molecules (omega-3 polyunsaturated fatty acids, lecithin), vitamins (folic acid, B6, B12, C, E, D3), minerals (chromium), and antioxidants (coenzyme Q10, glutathione, polyphenols, caffeine), as recently emphasised [17,18,19]. Furthermore, recent research pointed out that many plant-derived active compounds could be relevant in AD management [20], not restricted to diet intervention, but also addressing traditional medicine resources [21].

Thus, based on the current knowledge of AD pathophysiology, as well as based on the repeatedly documented anti-inflammatory and antioxidant potential of different functional foods and herbs, we aimed to find, describe, and correlate several dietary compounds that could be useful in formulating a nutritional approach in AD management.

## 2. Alzheimer’s Disease and Food—What Is the Correlation between Pathophysiology and Nutrition

Alzheimer’s disease is diagnosed according to DSM-V (Diagnostic and Statistical Manual of Mental Disorders, fifth edition [22]) or NIA-AA (National Institute on Aging and the Alzheimer’s Association [23]) mainly based on the displayed symptomatology associated with the insidious onset or gradual progression in memory, learning, language, visuospatial, and executive cognitive functions loss, and significant changes in behavioural and personality response, and on imaging and molecular relevant biomarkers confirmation (suggestive brain volume changes, Aβ deposits, and tau filaments presence, serological testing, and the presence of deterministic mutations detected by genetic testing).

From a physiological perspective, AD is mainly characterised by the progressive decline of cognitive functions alongside significant changes occurring in the ability to perform day-to-day tasks and in the overall capacity to relate and respond to external stimuli (socio-affective impairments and personality shifts). In contrast to the other two major neurodegenerative disorders, Parkinson’s disease (PD) and amyotrophic lateral sclerosis (ALS), AD pathophysiology mainly refers to altered cognitive abilities and only secondarily to impaired motor functions, suggesting that the disease mechanisms of action are slowly progressing throughout the years (having the onsets many years before the symptoms occur) debilitating brain parts in charge of neuronal plasticity (hippocampus), memory and perception (entorhinal cortex), speech, social interaction (cerebral cortex), and eventually automatic functions (brain stem) [24,25,26,27].

From a molecular point of view, extensive studies showed that AD molecular hallmarks are Aβ deposits (caused by aberrant amyloid precursor protein processing) and tau filaments (caused by tau protein hyperphosphorylation) that can be found to progressively accumulate in the mentioned brain areas [28,29]. As a result, the reduction in cerebral blood flow and the blood–brain barrier disruption led to neuronal pathways signalling and communication impairment and eventually to neuron loss [30,31,32].

Comprehensive molecular studies recently demonstrated the complex pathophysiology of AD, which include neuroinflammation and oxidative signalling, as being one of the missing links between histopathological features and disease molecular pathways [33]. In this way, reasonable evidence about the implications and role of inflammatory response in AD suggested that the main trigger for neuroinflammation is brain microglial macrophages chronic activation leading to pro-inflammatory cytokines sustained production (interleukin-1β, interleukin-6, and tumour necrosis factor-α) [34,35]. In this pathological molecular context, mitochondrial dysfunction and oxidative stress gain relevance and contribute to the molecular context fuelling the vicious cycle of oxidative stress-induced neuroinflammation [36,37].

As a response to the molecular pathological processes, including and mainly referring to neuroinflammation and oxidative stress, many recent studies reported several possible anti-inflammatory and antioxidant approaches that could contribute to slowing down or stopping AD progression [38,39]. In this context, despite how the impact of diet and nutrition on age-associated cognitive decline has not yet been fully described, nor the molecular pathways through which diet modulates cognition, it was reported that various minerals, micronutrients, vitamins with antioxidant/anti-inflammatory properties could be relevant in AD management. For instance, dietary essential fatty acids were previously described to be incorporated in neuronal membranes and to possess antioxidant, anti-excitotoxic, and anti-inflammatory potential [40,41,42]. In addition, their association with cognitive functions suggested that the synergistic interactions of different nutrients could be the key to their potential [43].

It was also shown that various nutrients that are predominant in several diets exhibit immune system modulation potential, being able to influence neuroinflammatory processes, as described in animal model studies [44,45]. Thus, polyphenols, unsaturated fats, and antioxidant vitamins could regulate oxidative balance and the neuroinflammatory response, while saturated fatty acids were described as brain tissue inflammation promoters [46,47].

In addition to these, it was shown that diets could significantly interfere with other components of the digestive system, such as the microbiota. In the case of AD, it was shown that a Mediterranean diet could modulate gut microbiota activity and diversity and further suggested that some microbiotic species could exhibit neuroprotective properties [48]. Recent reports regarding AD pathophysiology gave positive responses about the correlation between gut microbiota and impairments and brain function decline, suggesting that microbiome dysfunction could be a stable component of neurodegenerative pathologic mechanisms [49,50].

Additionally, compelling evidence shows that nutrients and other bioactive dietary compounds could influence neuroinflammatory processes leading to neurodegeneration in a synergistic manner for cumulative effect [51].

## 3. Vitamin E Odyssey and AD

Due to its lipophilic properties, the antioxidant vitamin E (α-tocopherol) could interact with cell membranes and interrupt the chain reactions resulting in neuronal injury by scavenging free radicals, chain-breaking antioxidants in lipoproteins and cell membranes, limiting lipid peroxidation, and maintaining membrane integrity [52]. The neuroprotective effects of vitamin E have been widely described in animal model studies with respect to its protective properties against cerebrovascular and neurodegenerative diseases.

Regarding its contribution to neuroprotection, several studies reviewed the potential of vitamin E to counteract oxidative stress induced by Aβ [53,54,55]. Yatin et al. [56] demonstrated that vitamin E was able to prevent Aβ_1–42_ induced protein oxidation, Aβ-induced ROS production, and accumulation leading to significant neuroprotective properties in rat embryonic hippocampal neuronal cell cultures. On the other hand, its neuroprotective potential could not be limited to its antioxidant properties, but vitamin E could also reduce the Aβ_1–42_-induced expression of glutamate transporter-1 (GLT-1), the main glutamate transport in mouse adult brains [57]. This interaction was described in mouse astrocytes as being mediated by oxidative stress leading to GLT-1 ubiquitination, a prolonged extracellular lifetime of released glutamate, and mislocalisation on the cell membranes surface, which were all prevented by vitamin E administration (water-soluble analogue, Trolox) [57] (Table 1).

The action of vitamin E was also tested against Aβ toxicity in rats, which were infused with Aβ_1–42_. Reference [57] showed that 3-day pre-treatment with oral vitamin E could prevent Aβ-induced learning and memory deficits. However, Yamada et al. [58] reported that the effects of vitamin E are independent of oxidative stress modulation in preventing Aβ toxicity, in a rat model of memory loss. On the other hand, several studies raised concerns about the possible toxicity of high doses of vitamin (≥400 IU/day) reported to increase the risk of mortality [59], while doses below 400 IU/day could be a preventive factor for all-cause mortality, as shown by meta-analysing 19 studies comprising more than 130,000 participants. Additionally, serious concerns could arise from the already demonstrated adverse effects of vitamin E unravelled by the stroke studies (i.e., increase in haemorrhagic stroke risk) that could impose supplemental risks to AD patients [60].

Yet, other studies failed to demonstrate this toxic effect [61], but reported that AD patients receiving up to 2000 IU vitamin E/day survived longer, as compared to those treated with cholinesterase inhibitors or with no drugs. Similarly, the beneficial effect of vitamin E supplementation against cognitive decline was also shown in women with low dietary intake of vitamin E (lower than 6.1 mg/day), but not compared with women receiving a high dietary intake of vitamin E [62] (Table 1).

Despite these positive results and possible concerns, vitamin E is not currently recommended for AD treatment or prevention [63]. Thus, two major causes could contribute to the previous negative results: (1) Wrong dose. The meta-analysis of Miller et al. suggested that more than 400 IU vitamin E/day could increase mortality; however, Lloret et al. [64] postulated vitamin E dose adjustment by correlation to plasmatic oxidised redox potential. (2) Wrong timing. When AD and MCI cognitive symptoms occur, the molecular and physiological processes are impaired; thus, many synapses are already lost, and many neurons are affected by neurofibrillary tangles at a faster rate than they can be replaced. In this way, the treatment with vitamin E could be ineffective in these stages [65], as some authors have already suggested that pre-symptomatic AD could be an efficient target for treatment, as many of the pathological processes could still be reversible [66,67,68].

**Table 1:** The correlation between AD and vitamin E neuroprotective potential.

Study	Study Type	Study Details	Intervention	Results
Animal models				
[52]	Review	-	-	○ Vitamin E induces chain-breaking antioxidant effects in lipoproteins and cell membranes. ○ Reduces lipid peroxidation. ○ Maintains membrane integrity.
[56]	RCS	Rat hippocampal cell culture	50 µM α-tocopherol	○ Vitamin E was able to prevent amyloid protein oxidation, ROS production, and neurotoxicity. ○ Aβ-induced free radicals scavenger.
[57]	RCS	Mouse astrocytes	100 µM Trolox	○ Aβ_1–42_ generated oxidative stress reduced surface expression of GLT-1 glutamate transporter. ○ Extracellular glutamate was increased due to GLT-1 ubiquitination and mislocalisation within the cellular membranes. ○ Trolox prevented the amyloid-induced effects.
[53]	RCS	Rats	333 IU/kg α-tocopherol	○ Vitamin E prevented learning and memory deficits when administrated three days before Aβ infusion (oral administration). ○ Aβ-treated rats did not show increased oxidative stress, and the antioxidant action of vitamin E was not demonstrated.
Human patients				
[59]	Meta-analysis	135,967 participants	≥400 IU/day vs. <400 IU/day Vit. E	○ High doses of vitamin E (≥400 IU/day) could increase mortality risk, as revealed by 9 out of 11 included trials analysing risk for all-cause mortality (risk difference > 0). ○ Trials included patients with variable degrees of morbidity, and the results may not generalise to healthy population.
[60]	RCT	613 participants	2000 IU/day α-tocopherol	○ Vitamin E reduced AD mortality compared to those receiving placebo.
[60]	RCT	847 participants	2000 IU/day α-tocopherol	○ 2000 IU/day vitamin E improved AD survivability compared to those treated with standard treatment or with no drugs.
[62]	RCT	6377 women	600 IU q.a.d. α-tocopherol	○ Vitamin E showed no significant modulation of cognitive functions compared with placebo. ○ Vitamin E’s positive effect on cognitive functions was more significant in groups where dietary vitamin E intake was low.
[61]	Review	-	-	○ Adverse effects of vitamin E unravelled in the stroke studies (i.e., increase in haemorrhagic stroke risk) may impose risks on AD patients.

## 4. Why Will Omega-3 Fatty Acids Not Be Enough?

Omega-3 polyunsaturated fatty acids (PUFAs) exhibit neuroprotective properties and represent a potential treatment for a variety of neurodegenerative and neurological disorders [69,70]. While triglycerides are energy metabolism substrates in low glucose states, PUFAs are membrane lipids providing structural and functional support. As high membrane fluidity is essential in maintaining synaptic integrity, PUFAs inclusion in neuronal membranes decreases the main membrane rigidity promoter: the total cholesterol fraction [71].

Three main PUFAs were intensely studied: eicosapentaenoic acid (EPA), docosapentaenoic acid (DPA), and docosahexaenoic acid (DHA). While EPA was frequently reported as beneficial in mood disorder improvement, attention to DHA was mainly attracted by its neuroprotective potential with respect to neurodegenerative conditions. DHA is quantitatively the most important omega-3 PUFA in the brain, whereas the availability of high-purity DPA preparations has been extremely limited until recently.

Omega-3 PUFAs exert pleiotropic effects on the cardiovascular and central nervous systems that were extensively correlated with age-related cognitive decline protection. Low omega-3 PUFA intake is one of many overlapping risk factors for both cardiovascular disease and AD and was also reported in diabetes, hypercholesterolemia, hypertension, hyperhomocysteinemia, dietary saturated fats, cholesterol, antioxidants, alcohol consumption, smoking, atrial fibrillation, and atherosclerotic disease [72]. While the cardiovascular protective effects of omega-3 PUFAs are backed by repeated positive clinical trial results, which lead to recommendations for dietary supplementation [73], the clinical trials for dementia prevention did not report suggestive results. Nevertheless, a 2005 literature evidence-based meta-analysis [74] on omega-3 PUFAs and dementia concluded that sufficient evidence to suggest the possible relevance of omega-3 PUFAs potential in the treatment and prevention of AD is yet available, but clinical trials should be the ones that would provide recommendations.

On the other hand, DHA could be a key structural and functional component of the brain’s memory consolidation areas. Moreover, high dietary intake resulted in increased hippocampal DHA levels, which have been shown to promote hippocampal-dependent learning processes [75]. Increased dietary intake of omega-3 PUFAs has been directly associated with increased grey matter volume in corticolimbic circuitry that represents the affective input for memory formation and cortical arousal in the brain [76].

Several studies reported that increased PUFA dietary intake could decrease dementia risk. The Canadian Study of Health and Aging (CSHA) rigorously examined plasma PUFA profiles cross-sectionally in a small cohort of 84 subjects [77] (Table 2). Plasma total PUFAs, DHA, and n-3/n-6 ratios were found to be decreased in AD, non-AD dementias, and cognitive impairment, but not in demented individuals. Yet, omega-6 PUFA levels were increased in AD and cognitively impaired patients, as compared to normal individuals and non-AD dementia patients, suggesting that decreased plasmatic PUFA levels could be a risk factor for pathologic cognitive decline. Barberger-Gateau and colleagues [78] reported administering FFQs (food-frequency questionnaires) to 1674 subjects and then following up for 2, 5, and 7 years. A total of 170 new cases of dementia were diagnosed during the follow-up period, with higher percentages in the group with low dietary intake of fish and seafood. Another case–control study replicated the CHSA methodology on 148 AD subjects and 45 cognitively normal controls [79]. Total serum saturated fatty acids, EPA, and DHA levels were significantly reduced in AD, as compared to normal individuals. However, their study managed to report that only serum total saturated fatty acids and DHA levels were associated with Mini-Mental State Examination (MMSE) and Clinical Dementia Rating scale (CDR) scores in stepwise multiple regression analyses (Table 2).

Thus, significant deficits of EPA and DHA were found in neurodegenerative disorders, but the studies’ limitations and heterogeneity prevent a clear conclusion. In this context, further investigation into the roles that individual polyunsaturated fatty acids have on brain health, protection, and repair will facilitate appropriate dietary recommendations and targeted therapeutic interventions.

## 5. Selenium and AD: Is There a Link?

**Figure 1:**
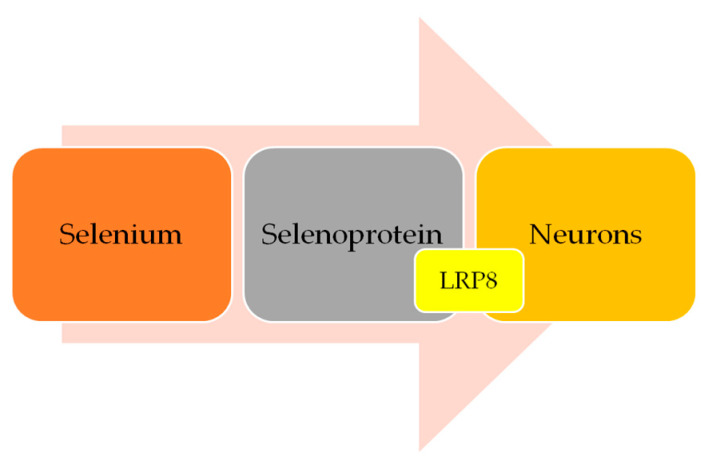
Brain selenium transport mechanism (simplified).

Selenium is a trace element crucial to cerebral functions. Following brain tissue selenium depletion, brain selenium is provided at the expense of other tissues. Severe selenium deficiency could lead to irreversible brain damage [80]. Selenium enters the neuron through the apolipoprotein E receptor 2 (LRP8), after being transported by selenoprotein P (Figure 1). The same protein has been found in high amounts in mice brains, where it was shown to be relevant for the maintenance of proper functions. SEPP knockout models develop severe neurological dysfunction, especially when fed a low selenium diet [81,82].

**Figure 2:**
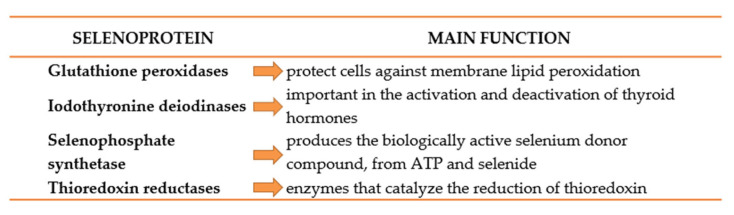
Main selenoprotein classes and their involvement in AD pathomechanism.

Selenium and selenoproteins (Figure 2) have been shown to have protective actions against cognitive decline, especially in patients with low dietary intake of selenium, as demonstrated by Shahar et al. [83], who found that performance associated with coordination tasks was associated with plasmatic selenium levels (1012 Italian participants aged 65 years or older). Serum selenium levels in AD were lower compared with MCI patients in a Spanish cohort [84]. Selenium supplementation (200 µg/day) was also found to reduce protein glycation through glycaemic control and modulating inflammatory response in an elderly Swedish cohort [85,86]. Similar results were obtained by Tamtaji et al. [87], with the addition that probiotics could potentiate selenium antioxidant proprieties and clinical effectiveness. Correlations between plasmatic selenium levels and cognitive decline have been found in populations with customary low dietary selenium intake, which may be responsible for the inconsistencies across the studies.

One of the mechanisms underlying the potential protective effects of selenium in AD may include inhibition of glycation and advanced glycation end products (AGE) formation with subsequent down-regulation of the AGE/AGE receptor pathway. The effects were shown to involve protein glycation inhibition in a dose-dependent manner. Furthermore, selenium influenced the interaction between amino acid residues with ROS scavenging and reduced α-carbonyl formation [88]. It is also notable that selenium may not only influence AGE formation but may also modulate AGE signalling and toxicity. Particularly, the role of selenium-induced inhibition of AGE formation in the prevention of p38 MAPK activation and subsequent COX-2 and P-selectin expression was demonstrated in human umbilical vein endothelial cells [89].

Selenium’s physiological effects were shown to be mediated and dependent on selenoproteins. Selenoproteins (GPX4, SELENOP, SELENOK, SELENOT, GPX1, SELENOM, SELENOS, and SELENOW) are the main ones expressed in the brain, especially in areas involved in AD; as such, they are promising targets for AD research [90]. Particularly, selenoprotein P (Sepp1) was found to be responsible for selenium transportation within mice brains and to be increasingly expressed by direct correlation to physical exercise. Using a Sepp1 knockout mouse model, Leiter et al. [91] demonstrated that selenium is responsible for hippocampal neurogenesis and in vitro neural cell proliferation, in a Sepp1-dependent manner. A recent trial found that selenium supplementation did not promote cognitive performance improvement in AD, while its CSF level was inversely correlated with MMSE scores after 24 weeks of treatment [92], these suggesting that selenium neuroprotective potential could be dependent on the specific transport protein expression (Table 3).

## 6. Traditional Medicine—How Would It Help?

To date, the Mediterranean diet has been shown to reduce the incidence of mild cognitive impairment (MCI) and, possibly, the conversion of MCI to dementia [93,94]. Additionally, it was shown that this contribution could be produced due to the increased content of vitamins, minerals, and antioxidants (such as polyphenols) that were positively associated with cognitive impairment prevention due to their antioxidant effects.

Sustained efforts are made to evaluate and compare the most-known diets’ effects on AD predisposition, prevention, and management [95]. However, Yusufov et al. [96] recently drew attention to the possible limitations of evaluating the effects of different diets on AD, as a much longer period of pathological processes remains clinically nonvisible (a dormant phase). In this context, it could be useful to consider a better understanding of the nutritional adjuvants as individual molecules rather than diets.

Thus, in addition to the nutritional intake of plants, there are traditional medicines that offer a variety of herbs that could be exploited as potent antioxidant sources. Plenty of studies address the potential of traditional medicines and herbs in cognitive decline and AD management; however, it was shown that polyphenols have the greatest potential compared to other molecule classes [97,98,99]. The potential of various plant extracts to modulate memory loss was previously described by our group [100], yet new resources are continuously found while the mechanisms of action of the most known are untangled.

*Ginkgo biloba* (*GB*) could be currently considered the oldest living tree species in the world and one of the best sources of cognitive performance improvers [101]. In traditional Chinese medicine, *GB* leaves were mainly used for the treatment of respiratory and cardiovascular disorders, while the seeds were frequently found efficient in pulmonary symptoms, alcohol abuse, and bladder infection treatments [102]. Currently, *GB* leaf-based extracts have numerous health benefits, including cognitive performance improvement [103].

In particular, it has been shown that *GB* extract could exhibit neuroprotective effects in both AD and vascular dementia (VD) based on the antioxidant, anti-inflammatory, and anti-apoptotic potential. Additionally, several studies showed that some *GB* extract components could be potent mitochondrial function modulators [104,105]. Other antioxidant effects of *GB* extracts are due to their capacity to modulate cerebral blood flow, neurotransmitter systems, cellular redox states, and nitric oxide synthesis [106,107]. In this context, a recent in vitro study using rat cerebellar granule cells showed that *GB* extract could successfully attenuate H_2_O_2_/FeSO_4_-induced oxidative damage resulting in efficient protection against oxidative stress-mediated apoptosis [108].

Most of the *GB* extracts’ antioxidant activity is the result of its flavonoid components’ biological effects: it contains kaempferol and quercetin, which modulate reactive oxygen species (ROS) metabolism in both in vitro and in vivo models [109], making *GB* extracts one of the most effective alternative solutions in cognitive decline and AD management. Shi et al. [110] thus described that the *GB* extract flavonoid fraction is responsible for the antioxidant effect through direct ROS scavenging, chelation of pro-oxidative heavy metal ions, and increased expression of antioxidant proteins, such as superoxide dismutase (SOD) and glutathione reductase (GSH). Additionally, the cytochrome P-450 enzyme system reducing the ROS formation and peroxide anion release could be another target of the *GB* extract flavonoid fraction [111].

Moreover, *GB* extracts were screened for the modulatory activity of the mitochondrial respiratory chain components (complexes I, IV, and V) in a study of senescent cells obtained from two age groups of mice with induced nitration stress [112]. However, the beneficial effects could only be seen in the cells obtained from older animals, which proved that the extract is effective against senescent cells.

The main mechanisms of memory and cognitive function improvement due to *GB* extract treatment could include increased blood flow in the brain, protective effect against peroxidation of brain lipids, easier utilization of oxygen and glucose by brain cells, reduction in amyloid plaque deposition [113], and lowering Aβ oligomers and APP levels [114]. Thus, *GB* derivatives have a proven beneficial effect against cognitive disorders, including mild and moderate AD. Unfortunately, the randomised trial reports regarding *GB* derivates’ clinical effects in AD are rather scarce. Despite its recognised potential in neurodegenerative disease treatment, *GB* derivates’ mechanisms of action and possible clinical application are still a widely controversial subject [115].

A significant compound belonging to the phenols class that exhibits great antioxidant potential and that was extensively studied in the AD context is curcumin, which is generally found in *Curcuma longa* extracts at 2 to 5% of dried mass [116]. Both *Curcuma longa* (*CL*) extracts and purified curcumin were studied for neuroprotective, antioxidant, and anti-inflammatory potential, despite that these aspects were long before suggested. In this way, *CL* extracts were found to protect the brain cells against oxidative stress and inflammatory processes driven by Aβ accumulation and, in contrast to *GB*, curcumin was shown to be implicated in Aβ plaque clearance while activating macrophages, microglia, and reactive astrocytes [117,118,119,120].

Another important active compound group that was extensively studied for neuroprotective potential is the ginsenosides found in *Panax ginseng* roots (*PG*). Guo et al. [20] recently meta-analysed some traditional Chinese medicine resources and found that ginsenosides are extensively used to prevent or slow cognitive decline. The main action of ginsenosides is to reduce Aβ neurotoxicity by preventing its production and accumulation in the brain in some animal models of AD, or to improve memory functions by inhibiting acetylcholinesterase activity (as recently discussed by [121,122]). Moreover, these aspects were then seen in AD patient trials, with promising results for finding a new and ground-breaking resource in AD management (Table 4).

## 7. Conclusions

Nutrients could be a key component in the physiologic and anatomic development of the brain. Several nutrients have been studied in the pursuit of the mechanism triggered by the pathology of AD: vitamin D, fatty acids, selenium, as well as neuroprotective plant extracts (i.e., *Ginkgo biloba*, *Panax ginseng*, and *Curcuma longa*), suggesting that the nutritional patterns could modulate the cognitive status and provide neuroprotection. The multifactorial origin of AD development and progression could suggest that nutrition could greatly contribute to the complex pathological picture. The identification of adequate nutritional interventions and mechanistic gaps in research could be the next steps in finding several innovative treatment options for neurodegenerative disorders.

## Figures and Tables

**Table 2:** The correlation between AD and fatty acids neuroprotective potential.

Study	Study Type	Study Details	Intervention	Results
[69]	Review	-	-	○ Deficits in DHA and EPA have been variably associated with neurodegenerative disorders. ○ The non-cognitively impaired elderly population benefit from DHA supplementation.
[72]	Review	-	-	○ CAD patients should consume 1 g/day DHA and EPA. ○ Patients with hypertriglyceridemia benefit from the consumption of 3–4 g/day EPA, DHA. Healthy subjects benefit from 250 to 500 mg/day.
[73]	Review	-	-	○ Normal aging: no association between omega-3 FA intake and cognitive loss. ○ Dementia: fish consumption correlates with the reduction in AD’s incidence; total omega-3 FA consumption correlates with reduced AD incidence; DHA correlates with reduced incidence for AD, while EPA does not. ○ Omega-3 FA supplementation improves MMSE scores in patients with dementia.
Animal models				
[74]	RCS	16 (8) Rats (omega-3 depleted)	300 mg/kg/day DHA	○ DHA group presented fewer reference memory errors compared with control in radial maze task, with no difference in working memory errors. ○ DHA group presented less MDA/mg protein compared with control in the cerebral cortex but not in the hippocampus.
Human patients				
[75]	Correlational study	55 participants	Self-reported consumption of omega-3 FA	○ Increased grey matter volume in the sgACC, right hippocampus, and amygdala in the high intake group.
[77]	Correlational study	84 participants (19 AD; 10 OD; 36 CIND; 19 Control)	FA analysis in blood plasma	○ Total unsaturated fatty acids, individual unsaturated fatty acids, and their fractions were lower across the spectrum of cognitively declined patients. Omega-6 fatty acids were higher in AD and CIND cases than in normal and non-AD dementias.
[78]	Longitudinal study	8085 healthy adults	-	○ The 7 years incidental dementia risk was lower in the group with high dietary intake of fish and seafood.
[79]	Correlational study	148 participants (29 M) Control: 45 (9 M)	FA analysis in blood plasma	○ DHA and total serum PUFA correlate with AD severity measured by MMSE and compared with healthy age-matched controls.

**Table 3:** The correlation between AD and selenium neuroprotective potential.

Study	Study Type	Study Details	Intervention	Results
[89]	Experimental study	Se nanoparticles on a bovine albumin and glucose medium		○ Selenium nanoparticles showed a dose-dependent inhibitory effect on glucose bonding and fructosamine formation.
[90]	Correlational study	Vascular endothelial cell cultures		○ Selenium reduces the expression of COX-2, P38 MAPK, and P-selectin in high-glucose, high-insulin, and advanced glycation end products’ rich cell cultures.
Animal models				
[91]	RCS	Mice	Normal cage vs. spin wheel cage	○ Exercise increases Selenoprotein P (Sepp1).
		Mouse neural cell culture	Selenium-controlled growth medium	○ Neural precursor cell formation from the hippocampal dentate gyrus and sub-ventricular area positively correlates with increasing Se concentration. ○ Newly developed cells’ diameter increases with Se concentration. ○ Sodium selenite promotes BetaIII-tubulin+ neuron differentiation.
		Mice	Sodium selenite infusion	○ NaSe infusion promotes hippocampal precursor proliferation, neurogenesis, and recruitment of neural stem cells. ○ Se decreases cellular reactive oxygen species in vivo and ex vivo.
		Mice	Sepp1 knockout	○ Sepp1 knockout model inhibits neural precursor cell proliferation and diameter. ○ Activity exerted by Sepp1 knockout mice was similar to wild type, although expressed less Sepp1.
		Mice	Endhotelin-1-induced hippocampal lesion	○ Selenium reverted the induced learning and memory deficits on Y-maze, novel object recognition, and fear conditioning tests.
Human patients				
[92]	RCT	40 probable AD patients	1 mg Na_2_SeO_4_/day vs. 10 mg Na_2_SeO_4_/day vs. placebo	○ Selenium supplementation reported no significant cognitive performance improvement compared with placebo after 24 weeks. ○ MMSE scores correlated with CSF Se levels, suggesting that the beneficial effects of selenium supplementation might be dependent on CSF retention.
[87]	RCT	79 patients with AD	200 μg /day Se vs. 200 μg /day Se + probiotics vs. placebo	○ Clinically insignificant and statistically significant improvement in MMSE scores in Se and probiotics branch compared with placebo. ○ Reduced CRP and increased glutathione in both active branches compared with placebo. ○ Increased total antioxidant capacity in Se + probiotics group compared with placebo. ○ No statistically significant changes in malondialdehyde levels.

**Table 4:** The correlation between AD and some traditional medicines herbs’ neuroprotective potential.

Study	Study Type	Study Details	Intervention	Results
Animal models				
[108]	Experimental study	Rat cerebellar granule cell culture	100 μg /mL *GB* extract	○ *GB* extract reduced TBARS formation by FeSO_4_ + H_2_O_2_ and improved cell survivability.
[109]	Experimental study	Rat cerebellar granule cell culture	100 μg /mL *GB* extract	○ *GB* extract reduced TBARS and LDH formation by FeSO_4_ + H_2_O_2_ and improved cell survivability. Cells treated with GB reduced the effects of FeSO_4_ + H_2_O_2_ Bcl-2 mRNA expression.
[112]	Experimental study	Rat and mice isolated mitochondria	10, 100, and 500 μg /mL *GB* extract	○ *GB* extract protected the mitochondria against nitric oxide, sodium nitroprussiate, and peroxide action, and yielded more ATP, as compared to control.
[113]	Experimental study	APP/PS1 mice	50 mg/kg *GB* extract *ad libitum*	○ *GB* extract inhibited the formation of Aβ plaques and pro-inflammatory interleukins mRNA expression (IL-1β, IL-6, TNF-α), and increased mRNA expression for anti-inflammatory IL (IL-4, IL-13, TGF-β).
[114]	Experimental study	TgAPP/PS1 mice	100 mg/kg *GB* extract *ad libitum*	○ *GB* extract promotes cell proliferation in the hippocampus dentate gyrus of transgenic mice and inhibits Aβ oligomer formation.
[122]	Experimental study	APP/PS1 double-TG mice	100 and 500 mg/kg/day, p.o. for 12 days, white *PG* powder	○ White ginseng reduced Aβ formation and improved memory functions.
[123]	Experimental study	ICR mice + 2 mg/kg, i.p. scopolamine	200 mg/kg, p.o., red, white, and black *PG* extracts	○ Ginseng extracts improve memory functions and cholinergic system functions by inhibiting acetylcholinesterase activity.
[124]	Experimental study	Sprague Dawley rats tauopathy model	Pre-treatment: 10 mg/kg for 7 days red *PG* extract	○ Red ginseng extract reduced tau protein hyperphosphorylation by specific phosphatase activities.
[119]	Experimental study	In vitro rat cells	4–20 mM curcumin	○ Curcumin decreased astrocytes proliferation, and improved oligodendrocytes proliferation.
Human patients				
[90]	Meta-analysis	1207 AD, VD	120–240 mg *GB* extract	○ *GB* extract yields improvement in cognitive, functional, and clinical ratings compared with placebo. The size of the effect was higher in studies using 240 mg of *GB* extract.
[120]	Meta-analysis	32 studies—human cells, animal cells, and animal models	0.1–200 μL extract/7.5–400 mg powder *CL*, 5 days–6 months	○ Antioxidant, anti-inflammatory, neuroprotective effects. ○ Improved behaviour (learning and memory).
[106]	Experimental study	Microglial cell culture	*GB* extract 10–90 μg/mL	○ The addition of *GB* extract over Aβ1–42 treated microglial cell culture improved cell viability. Moreover, *GB* reduced measured TNF-α and IL-1β and mRNA expression.
[107]	Experimental study	Human keratinocyte cell culture	1 mg/mL *GB* extract	○ *GB* preparation contains high amounts of quercetin and improved cell survivability in DPPH, ABTS, and AAPH-supplemented growth mediums.
[125]	Experimental study	AD patients	*PG* powder (4.5 g/d), 12 weeks	○ *PG* powder improved memory and learning in AD patients, but treatment cessation caused cognitive decline similar to initial state.
[126]	Experimental study	AD patients	Red Korean *PG* powder, 9 g/day, 12 weeks	○ Red Korean *PG* significantly improved memory functions and reduced typical AD impairments (measured by ADAS and CDR).
[119]	Experimental study	Human macrophages from AD patients	36.8 mg/mL curcuminoids	○ Curcuminoids promoted Aβ uptake by macrophages.

## Data Availability

Not applicable.
